# Extensive Non-Coding Sequence Divergence Between the Major Human Pathogen *Aspergillus fumigatus* and its Relatives

**DOI:** 10.3389/ffunb.2022.802494

**Published:** 2022-07-07

**Authors:** Alec Brown, Matthew E. Mead, Jacob L. Steenwyk, Gustavo H. Goldman, Antonis Rokas

**Affiliations:** ^1^ Department of Biological Sciences, Vanderbilt University, Nashville, TN, United States; ^2^ Vanderbilt Evolutionary Studies Initiative, Vanderbilt University, Nashville, TN, United States; ^3^ Faculdade de Ciências Farmacêuticas de Ribeirão Preto, Universidade de São Paulo, Ribeirão Preto, São Paulo, Brazil

**Keywords:** fungal genomics, evolution, *Aspergillus fumigatus*, non-coding region, evolutionary rate, virulence factor

## Abstract

Invasive aspergillosis is a deadly fungal disease; more than 400,000 patients are infected worldwide each year and the mortality rate can be as high as 50-95%. Of the ~450 species in the genus *Aspergillus* only a few are known to be clinically relevant, with the major pathogen *Aspergillus fumigatus* being responsible for ~50% of all invasive mold infections. Genomic comparisons between *A. fumigatus* and other *Aspergillus* species have historically focused on protein-coding regions. However, most *A. fumigatus* genes, including those that modulate its virulence, are also present in other pathogenic and non-pathogenic closely related species. Our hypothesis is that differential gene regulation – mediated through the non-coding regions upstream of genes’ first codon – contributes to *A. fumigatus* pathogenicity. To begin testing this, we compared non-coding regions upstream of the first codon of single-copy orthologous genes from the two *A. fumigatus* reference strains Af293 and A1163 and eight closely related *Aspergillus* section *Fumigati* species. We found that these non-coding regions showed extensive sequence variation and lack of homology across species. By examining the evolutionary rates of both protein-coding and non-coding regions in a subset of orthologous genes with highly conserved non-coding regions across the phylogeny, we identified 418 genes, including 25 genes known to modulate *A. fumigatus* virulence, whose non-coding regions exhibit a different rate of evolution in *A. fumigatus*. Examination of sequence alignments of these non-coding regions revealed numerous instances of insertions, deletions, and other types of mutations of at least a few nucleotides in *A. fumigatus* compared to its close relatives. These results show that closely related *Aspergillus* species that vary greatly in their pathogenicity exhibit extensive non-coding sequence variation and identify numerous changes in non-coding regions of *A. fumigatus* genes known to contribute to virulence.

## Introduction

Invasive aspergillosis (IA), a human disease caused by members of the fungal genus *Aspergillus*, is responsible for >400,000 cases worldwide per year with a mortality rate between 50-95% (Bongomin et al., 2017). More than 90% of IA cases are caused by *Aspergillus fumigatus*, with about a dozen other species such as *Aspergillus lentulus*, *Aspergillus thermomutatus*, and *Aspergillus udagawae* accounting for the rest (Steinbach et al., 2012; Rokas et al., 2020). Studies in both environmental (Flores et al., 2014) and hospital settings (Wirmann et al., 2018) show that asexual spores (conidia) of *A. fumigatus* and many other *Aspergillus* species are present in the air, yet *A. fumigatus* causes IA more frequently than its close relatives.

IA begins with inhalation of *Aspergillus* asexual spores and subsequent interaction between the asexual spores and the epithelium of the lung (Chotirmall et al., 2013). Several defense mechanisms including physical removal of asexual spores (Croft et al., 2016), secretion of antimicrobial peptides (Wiesner and Klein, 2017), and recruitment of specialized immune cells are employed by the human host to prevent spore germination (Bertuzzi et al., 2018). To cause infection, *A. fumigatus* must overcome these challenges and adapt to the host environment. The dynamics and intricacies of the interaction between *A. fumigatus* and host responses have yet to be fully elucidated. Decades of work have identified at least 206 genetic determinants of *A. fumigatus* virulence, that is genes whose deletion is known to modulate the virulence of *A. fumigatus* (for a detailed list, see: Steenwyk et al., 2021a). These genetic determinants of virulence are involved in a wide range of activities including gene regulation, RNA processing, protein modification, production of secondary metabolites, amino acid biosynthesis, cell cycle regulation, morphological regulation, and others (Steenwyk et al., 2021b).

The phylogeny of the genus *Aspergillus* reveals that pathogenic species are often more closely related to nonpathogenic species than to other pathogenic ones (Steenwyk et al., 2009; Houbraken et al., 2014; de Vries et al., 2017; Rokas et al., 2020; Mead et al., 2021). For example, *A. fischeri* is a close relative of *A. fumigatus* (the two share >90% average nucleotide sequence similarity and >95% average amino acid sequence similarity between orthologs), yet *A. fischeri* is less virulent and is not considered clinically relevant (Mead et al., 2019; Steenwyk et al., 2020a). Given the large disparity of IA cases caused by *A. fumigatus* and closely related species, early studies looked to species-specific genes in *A. fumigatus* as a potential contributor (Fedorova et al., 2008). However, a recent examination found that 206 known genetic determinants of virulence in *A. fumigatus* are shared between *A. fumigatus* and at least one other closely related species (Mead et al., 2021).

Variation in non-coding regions can also contribute to phenotypic diversity (Carroll, 2008; Li and Johnson, 2010) and disease (Ropero et al., 2017; Jang et al., 2018; Caron et al., 2019). In fungi, non-coding regions found immediately upstream of genes’ protein-coding regions are bound by transcription factors (TFs), impact transcriptional activity (Kim et al., 2019), and play roles in vital biological processes such as zinc homeostasis (Eide, 2020) and thermotolerance (Yamamoto et al., 2008). Differences in gene expression have become an important focus in understanding *A. fumigatus* virulence (Chung et al., 2014; de Castro et al., 2014; Furukawa et al., 2020; Ries et al., 2020; Takahashi et al., 2021; Colabardini et al., 2022). However, the role non-coding regions play in differential gene regulation between *A. fumigatus* and close relatives remains largely unknown.

Here, we perform genome-wide comparisons of intergenic, non-coding regions upstream of the first codon of single-copy orthologous genes of the reference strains *A. fumigatus* Af293 and A1163 against those of eight closely related species. We identified 5,215 single-copy orthologous genes across the 10 taxa of interest. Of the 5,215 genes, the non-coding regions of 4,483 genes either lacked homology across the ten taxa or showed extensive sequence variation, such that multiple sequence alignment was not possible. For the remaining 732 genes, each non-coding sequence was ≥500 bp long in all ten taxa and the sequence similarity of the sequence alignment between the *A. fumigatus* Af293 sequence and those of all other nine strains/species was ≥75%, enabling us to construct accurate multiple sequence alignments. Examination of the evolutionary rates of the non-coding and protein-coding regions of these 732 genes identified 418 upstream non-coding and 100 protein-coding regions whose evolutionary rate was different in *A. fumigatus* compared to close relatives. These 418 non-coding regions include 25 known genetic determinants of *A. fumigatus* virulence, such as *pkaR* (a regulatory subunit essential for protein kinase A pathway), *gliG* (glutathione S-transferase required for gliotoxin production), and *metR* (transcription factor required for sulfur assimilation).

## Methods

### Genomic Data Collection

All *Aspergillus* genomes are publicly available and were downloaded from NCBI (https://www.ncbi.nlm.nih.gov/). These strains include *A. fumigatus* Af293 (Nierman et al., 2005), *A. fumigatus* A1163 (Fedorova et al., 2008), *A. oerlinghausenensis* CBS139183 (Steenwyk et al., 2020b), *A. fischeri* NRRL1881 (Fedorova et al., 2008), *A. lentulus* IFM54703 (Kusuya et al., 2016), *A. novofumigatus* IBT 16806 (GenBank accession: MSZS00000000.1) *A. fumigatiaffinis* 5878 (dos Santos et al., 2020), *A. udagawae* IFM 46973 (Kusuya et al., 2016), *A. turcosus* HMR AF 1038 (Parent-Michaud et al., 2019), and *A. thermomutatus* HMR AF 39 (Parent-Michaud et al., 2019).

### Identification of Single-Copy Orthologous Genes

To infer single-copy orthologous genes among all protein-coding sequences for all ten taxa, we used OrthoFinder, version 2.4.0 (Emms and Kelly, 2015). OrthoFinder clustered genes into orthogroups from gene-gene sequence similarity information obtained using the program DIAMOND version 2.0.9 (Buchfink et al., 2015) with the proteomes of the ten *Aspergillus* species as input. The key parameters used in DIAMOND were e-value = 1 x 10^-3^ with a percent identity cutoff of 30% and percent match cutoff of 70%. This approach identified 5,215 single copy orthologous genes (**Table S1**).

### Identification of Highly Conserved Non-Coding Regions

To identify highly conserved non-coding regions, we first retrieved intergenic sequences directly upstream of the first codon of all 5,215 single-copy orthologous genes for each of the ten *Aspergillus* species/strains using a custom script (https://github.com/alecbrown24/General_Bio_Scripts; this script was based on a previously available script: https://github.com/shenwei356/bio_scripts). We retrieved the first 500 bp of intergenic sequence directly upstream of each gene’s first codon and used these sequences to generate FASTA files of non-coding regions, as well as FASTA files of single-copy orthologous protein-coding sequences using Python version 3.8.2 (https://www.python.org/). For some of the non-coding regions, there were <500 bp of non-coding sequence between the first codon of the gene of interest and an upstream gene; in these instances, only the intergenic region was used for subsequent analyses.

All multiple sequence alignments were constructed using MAFFT, version 7.453, with default parameter settings (Rozewicki et al., 2019). Analyses were conducted using custom Python scripts that used BioPython, version 1.78 (Cock et al., 2009), and NumPy, version 1.20.3 (Harris et al., 2020), modules. Sequence similarity in protein-coding and non-coding regions was calculated from their corresponding multiple sequence alignment files. The percent sequence similarity for each position in the alignment was calculated by determining if the nucleotide/amino acid at each position was the same as the nucleotide/amino acid for *A. fumigatus* Af293 and then dividing by 10. The percent similarity for each nucleotide/amino acid in each of the 5,215 non-coding and protein-coding regions of genes was averaged and reported. We discovered that the non-coding regions of only 732 of the 5,215 genes contained ≥500 bp of sequence directly upstream of the first codon in all ten taxa and exhibited sequence similarity ≥75% between the *A. fumigatus* Af293 sequence and each of the other nine strains/species, enabling us to construct accurate multiple sequence alignments. Thus, we focused our analyses on these 732 genes.

### Phylogenetic Tree Inference and Comparisons

To construct a phylogenomic data matrix, codon-based alignments for all 5,215 single-copy protein-coding orthologs were individually trimmed using ClipKIT, version 1.1.5 (Steenwyk et al., 2020a), with the ‘gappy’ mode and the gaps parameter set to 0.7. The resulting trimmed codon-based alignments were then concatenated into a single matrix with 9,248,205 sites using the ‘create_concat’ function from PhyKIT, version 1.2.1 (Steenwyk et al., 2021a). Next, the evolutionary history of the ten *Aspergillus* genomes was inferred using IQ-TREE, version 2.0.6 (Minh et al., 2020), and the “GTR+F+I+G4” model of sequence evolution, which was the best fitting one according to the Bayesian Information Criterion (Waddell and Steel, 1997; Vinet and Zhedanov, 2011). Bipartition support was assessed using ultrafast bootstrap approximations (Hoang et al., 2018). All bipartitions received full support. The inferred topology is congruent with known relationships inferred from analyses of single or a few loci as well as from genome-scale analyses (Steenwyk et al., 2009; Hubka et al., 2018; dos Santos et al., 2020).

To identify gene trees whose phylogeny was statistically different from the species phylogeny, we used the approximately unbiased test (Shimodaira, 2002). Protein-coding region and non-coding region trees were inferred using IQ-TREE, version 2.0.6 (Minh et al., 2020), with “GTR+I+G+F” as it was the best fitting substitution model (Waddell and Steel, 1997; Vinet and Zhedanov, 2011). The distributions of branch lengths for protein-coding region and non-coding region trees were determined using the “total_tree_length” function from PhyKIT version 1.2.1 (Steenwyk et al., 2021b) (**Table S2**).

### Analysis of Molecular Evolutionary Rates of Protein-Coding and Non-Coding Regions Between the Major Pathogen *A. Fumigatus* and its Relatives

To determine the rate of sequence evolution in protein-coding region alignments between *A. fumigatus* and close relatives, we examined variation in the ratio of the rate of nonsynonymous (dN) to the rate of synonymous (dS) substitutions (dN/dS or ω) across the phylogeny. We first obtained codon-based alignments from their corresponding protein sequence alignments using pal2nal, version 14 (Suyama et al., 2006). We next used the codon-based alignments to calculate ω values under two different hypotheses using the codeml module in paml, version 4.9 (Yang, 2007). For each gene tested, the null hypothesis (H_0_) was that all branches of the phylogeny exhibit the same estimated ω value. We compared H_0_ to an alternative hypothesis (H_A_) which allows for the branch leading to *A. fumigatus* to have a distinct estimated ω value from the rest of the branches. To determine whether H_A_ was significantly different from H_0_ for each of the codon-based alignments, we used the likelihood ratio test with a statistical significance threshold of α = 0.01.

To determine the rate of sequence evolution in non-coding region alignments between *A. fumigatus* and close relatives, we examined variation in the ratio of the rate of substitutions in each non-coding region (dNC) to the rate of synonymous (dS) substitutions in its corresponding protein-coding region (dNC/dS or ζ) across the phylogeny. Like the analysis of the protein-coding regions, the null hypothesis (H_0_) was that all branches of the phylogeny exhibit the same estimated ζ value. We compared H_0_ to an alternative hypothesis (H_A_) which allows for the branch leading to *A. fumigatus* to have a distinct estimated ζ value from the rest of the branches. ζ values were calculated under the different hypotheses using HyPhy version 2.2.2 (Pond et al., 2004) with the “nonCodingSelection.bf” batch file as established by Oliver Fedrigo (Haygood et al., 2007; Fedrigo et al., 2011). To determine whether H_A_ was significantly different from H_0_ for each of the non-coding region alignments, we used the likelihood ratio test with a statistical significance threshold of α = 0.01.

### Functional Enrichment Analyses of Genes With Signatures of Different Evolutionary Rates

To determine whether genes with signatures of different evolutionary rates in either their protein-coding or non-coding regions are enriched for particular functional categories, we implemented the Gene Ontology (GO) Term Finder webtool on AspGD (Cerqueira et al., 2013) using default settings. We conducted two separate analyses. The first examined those *A. fumigatus* genes that exhibited a different evolutionary rate in their non-coding regions, whereas the second examined those *A. fumigatus* genes with a different evolutionary rate in their protein-coding regions. These gene sets were compared to a general background set that includes all the features/gene names in the database with at least one GO annotation for *A. fumigatus*. Both analyses used a p-value cutoff of 0.05.

### Examination and Visualization of Mutational Signatures

To identify interesting examples of sequence variation between *A. fumigatus* and the other species for non-coding regions of genes of interest, we visualized and compared multiple sequence alignments using the MView function in EMBL-EBI (Madeira et al., 2019). Workflow of methods can be seen in **Figure S1**.

## Results

### *Aspergillus* Species Exhibit Extensive Sequence Variation in Their Non-Coding Regions

**Figure 1:**
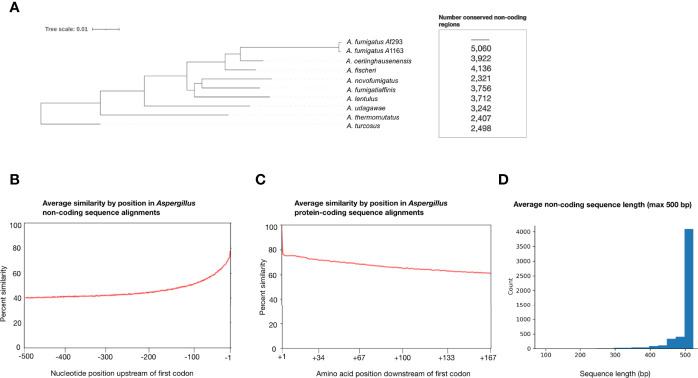
*Aspergillus* section *Fumigati* species exhibit sequence variation in non-coding regions that are 500 base pairs upstream of genes’ first codon. **(A)** Species phylogeny of two *A fumigatus* reference strains (Af293 and A1163) and closely related *Aspergillus* section *Fumigati* species constructed from concatenation analysis of a 5,215-gene data matrix. Branch lengths correspond to nucleotide substitutions/site. Note the long branch leading to *A fumigatus*, indicative of a greater number of nucleotide substitutions per site in this species. The number of genes whose non-coding regions were conserved (≥ 75% sequence similarity between each species and ≥ 500 bp in length) between *A. fumigatus* Af293 and each species are shown next to the corresponding taxa. **(B)** Average percent sequence similarity of non-coding regions of 5,215 genes by position, relative to the gene’s first codon. Sequence alignments of non-coding regions were compared by position and the average percent similarities for each site are reported with -1 indicating the site directly upstream of the first codon and -500 indicating the site 500 bp upstream of the first codon. **(C)** Average percent sequence similarity by position of the first 167 amino acid sites in the alignments of 5,215 genes, relative to the gene’s first codon. The average percent similarity for each site is reported, with +1 indicating the first amino acid. **(D)** Average sequence alignment lengths of the 5,215 non-coding regions examined in this study. 4,079 of the 5,215 non-coding regions have ≥ 500 bp in all 10 strains/species used in this study.

To analyze the sequence diversity of non-coding regions in section *Fumigati*, we first identified 5,215 single-copy orthologous genes amongst ten strains/species in the section (**Figure 1A**). We then computed the percent similarity between the non-coding and protein-coding regions of each *A. fumigatus* Af293 ortholog and their homologous non-coding and protein-coding regions in the other nine taxa (**Tables S3**, **S4**). Those individual percent similarities were then averaged to get the final percent similarity for the non-coding and protein-coding regions of that ortholog. Averaging the non-coding region percent similarities for the 5,215 single-copy orthologous genes revealed an average similarity of ~72%; 648 alignments exhibited <50% similarity, 3,665 exhibited sequence similarity between ≥50% and <75%, and 902 exhibited ≥75% similarity. Interestingly, three genes exhibited > 90% similarity. These genes were *cnaB*, whose protein product is a calcineurin regulatory subunit and whose transcript is induced by exposure to human airway epithelial cells (Juvvadi et al., 2011; Oosthuizen et al., 2011), *AFUA_6G07800*, which is predicted to be a transcription factor with unknown function (Cerqueira et al., 2013), and *AFUA_6G04530*, which is predicted to have a role in histone acetylation (Cerqueira et al., 2013).

Average percent similarity by position in *Aspergillus* non-coding region alignments (**Figure 1B**) revealed that the percent similarity directly upstream of the first codon (-1 bp upstream) is higher than 60% and decreases as the distance from the first codon increases, approaching 40% similarity. This result suggests that potentially conserved promoter and *cis*-regulatory elements occur in these non-coding regions and is consistent with transcription factor binding location in *A. fumigatus* (Chung et al., 2014; de Castro et al., 2014). For comparison, we also calculated the average percent similarity by position in the protein-coding region alignments of all 5,215 genes (**Figure 1C**). We found that the percent similarity of the first amino acid (+1) was ~100%, indicative of the first methionine; similarity was high throughout the first 167 sites of amino acid alignment but decreased as the distance from the first amino acid increased, approaching 60% amino acid sequence similarity.

Examination of the 4,567 genes whose non-coding region alignments exhibited ≥50% similarity (i.e., the 3,665 genes whose similarity was ≥50% and <75%, and the 902 that had ≥75% similarity) revealed several instances in which one or more sequences were poorly aligned for stretches of 100 bp or more. This was especially true when sequences in these alignments exhibited large variation in their lengths. Further, we found a low level of synteny as genes immediately upstream of a non-coding region of interest generally differed between species.

We also determined the number of conserved non-coding regions with ≥75% sequence similarity and that were ≥500 bp in length between *A. fumigatus* Af293 and each of the other nine taxa, separately (**Figure 1**). We found that *A. novofumigatus* shares the fewest number of conserved non-coding regions (2,321) despite being more closely related to *A. fumigatus* than other species included in our phylogeny (Steenwyk et al., 2009; Houbraken et al., 2014; Rokas et al., 2020); this suggests that the quality of annotations may differ across the ten genomes examined and that improvements in the gene annotation of these genomes could increase the number of conserved non-coding regions shared by these taxa. *A. fumigatus* A1163 shared the greatest number of conserved non-coding regions with *A. fumigatus* Af293 (5,020). With the exceptions of *A. novofumigatus*, *A. oerlinghausenensis*, and *A. thermomutatus*, the closer a relative is to *A. fumigatus* Af293, the greater the number of conserved non-coding regions that are shared.

### Phylogenetic Analyses Reveal Differences Between Non-Coding Region Trees and Protein-Coding Region Trees

To help determine if differences existed between non-coding and protein-coding regions across our species, we first compared total branch lengths in phylogenetic trees constructed from both non-coding and protein-coding regions from all 5,215 genes (**Figure S2**). Comparisons of the overall distributions between these two groups revealed a statistically significant difference between the overall branch lengths of protein-coding and non-coding regions (Wilcoxon signed-ranked test; p-value = 0.004), suggesting that non-coding regions of single-copy orthologs evolve faster than protein-coding regions.

### Many Non-Coding but Fewer Protein-Coding Regions Exhibit Different Rates of Evolution in *A. fumigatus*

**Figure 2:**
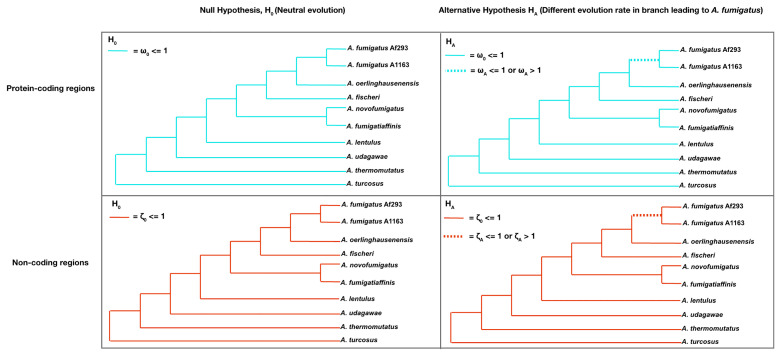
Examining whether the non-coding and protein-coding regions of 732 genes have different evolutionary rates in the major pathogen *A. fumigatus*. The top two panels present the null and alternative hypotheses for evolutionary rate difference in the protein-coding regions of *A. fumigatus* genes relative to the other species. The null hypothesis (H_0_, upper left) constrains all ω (dN/dS) values across all branches to be less than or equal to 1, the neutral evolutionary rate. The alternative hypothesis (H_A_, upper right) allows the branch leading to *A. fumigatus* (dashed branch) to have an ω value lower than, equal to, or greater than 1 (indicative of evolutionary rate difference) compared to the background branches. The bottom two panels present the null and alternative hypotheses for evolutionary rate difference in the non-coding regions of *A. fumigatus* genes relative to other species. Similarly, the null hypothesis (H_0_, bottom left) constrains all ζ (dNC/dS) values across all branches to less than or equal to 1. The alternative hypothesis (H_A_, bottom right) allows for the branch leading to *A. fumigatus* (dashed branch) to have a ζ value lower than, equal to, or greater than 1 (evolutionary rate difference) compared to the background branches. For each protein-coding and non-coding region, a likelihood ratio test was used to determine which hypothesis best fits the data.

Given the uncertainty regarding the homology of some sequences in these 5,215 non-coding region alignments and our finding that most sequence conservation was found near the first codon position, we focused our evolutionary rate analyses only on the 732 non-coding region alignments whose sequences were all ≥500 bp long and exhibited ≥75% sequence similarity between *A. fumigatus* Af293 and each other strain/species in the phylogeny. Briefly, to determine the rate of sequence evolution in protein-coding and non-coding region alignments between *A. fumigatus* and close relatives, we examined variation in the ratio of the rate of nonsynonymous (dN) to the rate of synonymous (dS) substitutions (ω value) for protein-coding regions and the variation in the ratio of the rate of non-coding (dNC) to the rate of synonymous (dS) substitutions (dNC/dS or ζ value) for non-coding regions across the phylogeny. To test whether the molecular evolutionary rates of protein-coding and non-coding regions differed between the major pathogen *A. fumigatus* and its relatives, we statistically examined whether protein-coding and non-coding *A. fumigatus* sequences evolved at a similar (H_0_) or different (H_A_) rate as those of other taxa (**Figure 2**).

**Figure 3:**
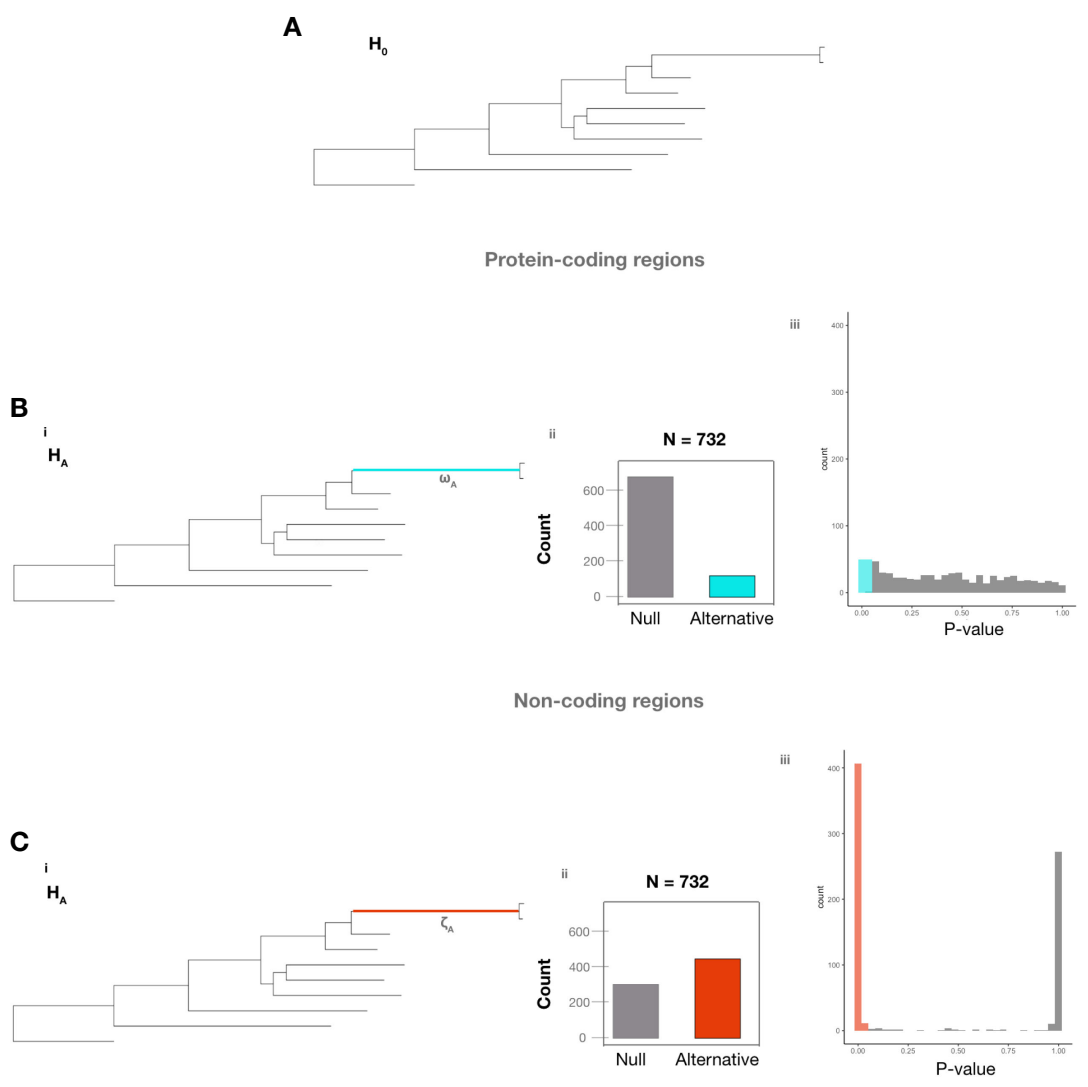
Non-coding regions of *A. fumigatus* genes exhibit many more signatures of evolutionary rate difference than their corresponding protein-coding regions. **(A)** The null hypothesis (H_0_) that all branches have the same evolutionary rate. **(B, C)** The alternative hypotheses assume that the ω value (Bi) or the ζ value (Ci) in the branch leading to *A. fumigatus* differs from the value in the rest of the branches of the phylogeny. Bii. 632 of 732 protein-coding regions (84.34%) did not reject H_0_ (gray) and 100 of 732 (16.66%) rejected H_0_ (blue). Biii. The distribution of p-values for protein-coding regions that did not (gray) and did (blue) reject H_0_. Cii. 314 of 732 non-coding regions (42.90%) did not reject H_0_ (gray) and 418 of 732 non-coding regions (57.10%) rejected H_0_ (red), which suggests a greater amount of variation in non-coding regions than in protein-coding regions between *A. fumigatus* and relatives. Ciii The distribution of p-values for non-coding regions that did not (gray) and did (red) reject H_0_.

Examination of protein-coding regions identified 100/732 genes (**Table S5**) (13.7% of examined genes) that significantly rejected H_0_ (under which all branches exhibited the same ω value) (**Figure 3A**) over H_A_ (which postulates that the ω value of the branch leading to *A. fumigatus* was distinct from the background ω value of all other branches) (**Figure 3B**). Examination of non-coding regions identified 418/732 genes (**Table S6**) (57.1% of examined genes) that significantly rejected H_0_ (under which all branches exhibited the same ζ value) over H_A_ (which postulates that the ζ value of the branch leading to *A. fumigatus* was distinct from the background ζ value of all other branches) (**Figure 3C**). Taken together, these results suggest a much higher amount of variation in non-coding regions than in protein-coding regions between *A. fumigatus* and relatives. The p-value distribution of protein-coding regions is uniform, while the p-value distribution of non-coding regions is bimodal with nearly all p-values being either under 0.05 or 1.0. This result suggests that the 418 non-coding regions exhibited major differences in their relative fit for the two hypotheses, whereas protein-coding regions exhibited much smaller differences.

### Genes With Signatures of a Different Evolutionary Rate in Non-Coding Regions are Enriched for Regulatory Functions in *A. fumigatus*

To identify functions that were over-represented in the list of genes that rejected H_0_ for either their coding or non-coding regions, we conducted gene ontology (GO) enrichment analyses. Examination of significantly over-represented GO terms for the 418 genes (**Table S7**) with signatures of different evolutionary rates in non-coding regions revealed numerous biological processes related to regulation, metabolism, and development (e.g., “cellular component organization or biogenesis”, p = 0.00016; “regulation of protein metabolic process”, p = 0.00101; “hyphal growth”, p = 0.00352; “regulation of cell cycle”; p = 0.0076; “developmental process”, p = 0.01233; “reproduction”. p = 0.00361). Of the 418 genes, 71 lacked any functional GO annotation. In comparison, for the 314 genes that did not exhibit a different evolutionary rate in their non-coding regions (**Table S8**), the only term that was enriched was “nucleotide binding” (p = 0.06129) and was found associated with 48 genes. For the 100 genes with signatures of different evolutionary rate in their protein-coding regions, only one function was found enriched (“regulation of cellular process”, p = 0.02647). Of note, 74 of the protein-coding genes lacked any functional GO annotation (**Table S9**).

### Four Genes Whose Non-Coding Regions Exhibit Different Evolutionary Rates in *A. fumigatus* also bind transcription factors That are Known Genetic Determinants of Virulence

**Figure 4:**
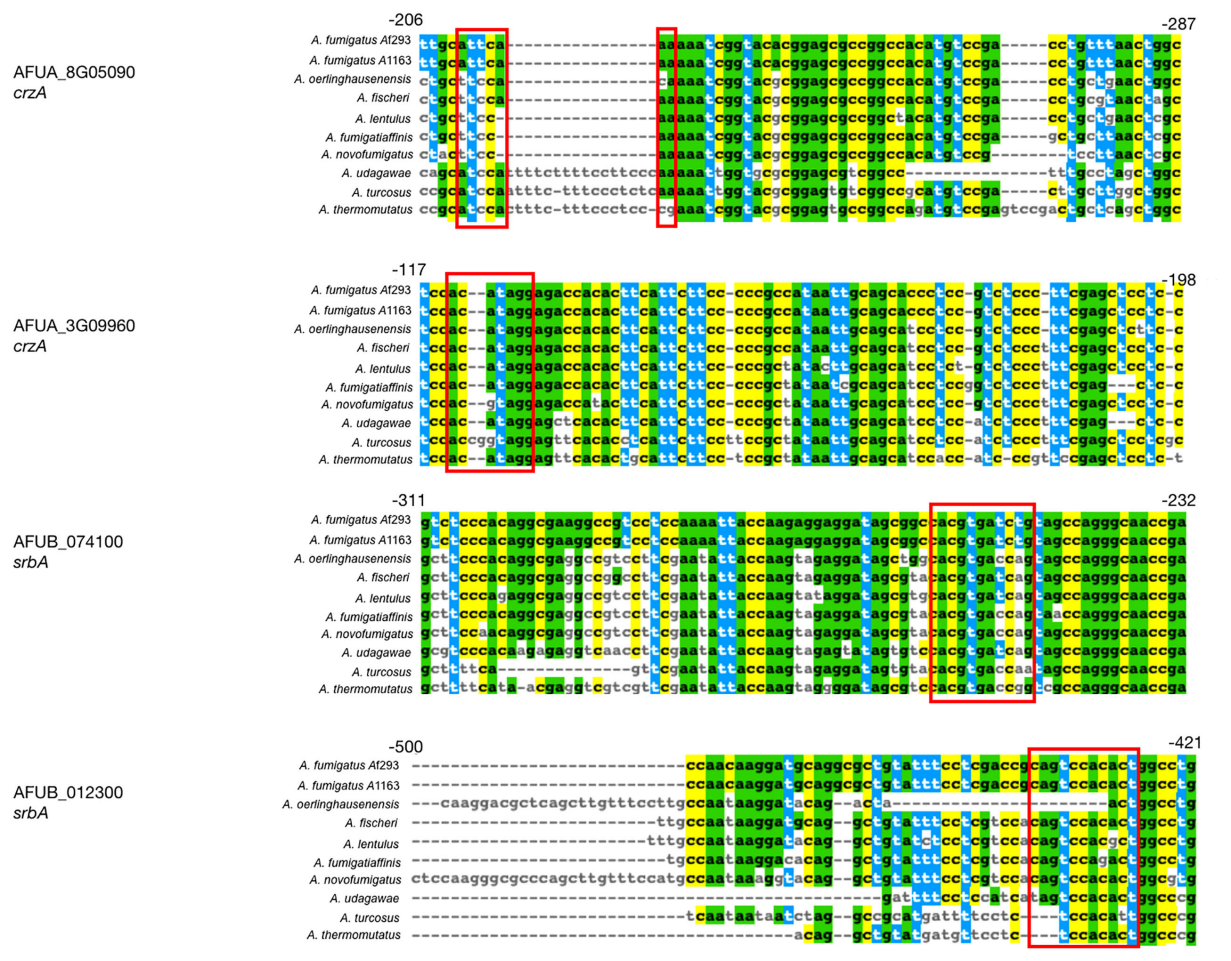
CrzA and SrbA binding locations in 4 genes that exhibit a different evolutionary rate in their non-coding regions. Two *A. fumigatus* genes (*AFUA_8G05090* and *AFUA_3G09960*) known to bind CrzA in their non-coding regions and two *A. fumigatus* genes (*AFUB_074100* and *AFUB_012300*) known to bind SrbA in their non-coding regions have at least a 2 bp difference in the TF binding site between *A. fumigatus* and one or more relatives. Red boxes represent the binding site locations found in previous ChIP-seq experiments.

To identify if any of the *A. fumigatus* genes with different evolutionary rates in their respective non-coding regions also contain known TF binding sites, we compared the list of 418 non-coding regions to binding sites of two TFs known to be genetic determinants of virulence, CrzA and SrbA (Cramer et al., 2008; Willger et al., 2008). ChIP-seq analysis of CrzA (de Castro et al., 2014) uncovered 110 genes that are directly bound by the TF in *A. fumigatus* strain Af293. Of these, 28 were reported to exhibit CrzA binding within 500 bp of the first codon, and two genes, *AFUA_8G05090* (a putative MFS transporter) and *AFUA_3G09960* (Aureobasidin resistance protein), exhibited a different evolutionary rate in the non-coding regions of *A. fumigatus* strains in our analysis. ChIP-seq analysis of SrbA (Chung et al., 2014) revealed 112 genes directly bound by the TF in *A. fumigatus* strain A1163. Of these, 57 were reported to exhibit SrbA binding within 500 bp of the first codon, and two genes, *AFUB_074100* (a gene of unknown function which appears to interact with *sldA*, a checkpoint protein kinase) and *AFUB_012300* (a gene predicted to be involved in nitrate assimilation), exhibited a different evolutionary rate in the non-coding regions of *A. fumigatus* strains in our analysis. Importantly, we found that for all four genes (*AFUA_8G05090*, *AFUA_3G09960*, *AFUB_074100*, and *AFUB_012300*) there was at least a 2 bp sequence difference between *A. fumigatus* and at least one close relative in their sequences at the TF binding site location (**Figure 4**). Together, our results suggest that intergenic non-coding regions that bind known TFs can exhibit substantial differences in their evolutionary rates between *A. fumigatus* and close relatives, which raises the hypothesis that these differences may lead to differences in gene expression.

### Non-Coding Regions Upstream of Genetic Determinants of Virulence With Different Rates of Evolution in *A. fumigatus*

We identified 25 genetic determinants of virulence whose non-coding regions exhibited a different rate of evolution in *A. fumigatus* (**Table 1**). Three genes (*metR, his3*, and *met16*) are involved in amino acid biosynthesis, eight genes (*chsF, calA, gel2, nrps1, gfa1, csmB, rlmA*, and *rodA*) are involved in cell wall biosynthesis, nine genes (*noc3, spe2, gus1, pri1*, *AFUA_2G10600*, *mak5, pkaR, ramA*, and *somA*) are involved in cellular metabolism, two genes (*aspB* and *tom40*) are involved in hyphal growth, and three genes (*gliG, gliI*, and *gliJ*) are involved in gliotoxin biosynthesis. Of the 25, 14 genes (*metR, chsF, calA, gel2, nrps1, gfa1, csmB, rlmA, rodA, AFUA_2G10600*, *pkaR, ramA, somA*, and *aspB*) have been shown to modulate virulence in an animal model of infectious disease. Three genes (*gliG, gliI*, and *gliJ*) are required for the biosynthesis of gliotoxin, a secondary metabolite involved in *A. fumigatus* virulence (Brakhage and Langfelder, 2002; Dagenais and Keller, 2009), and deletions of the eight remaining genes (*his3, met16, noc3, spe2, gus1, pri1, mak5*, and *tom40*) have been previously shown to be important for viability and therefore, likely virulence (for a detailed list, see: Steenwyk et al., 2021a). Interestingly, only two of these 25 genes (*csmB* and *rodA*) exhibit a signature of different evolutionary rate in their protein-coding region as well, a finding consistent with our result that there are more changes in non-coding regions than in protein-coding regions of *Aspergillus* genes.

**Table 1:** Twenty-five genetic determinants of *A. fumigatus* virulence have a different evolutionary rate in their non-coding regions.

Gene ID	Gene Name	Specific Function	General Pathway	Reference
*AFUA_4G06530*	*metR*	putative bZip transcription factor	amino acid biosynthesis	Amich et al., 2013
*AFUA_6G04700*	*his3*	Putative imidazoleglycerol-phosphate dehydratase	amino acid biosynthesis	Hu et al., 2007
*AFUA_3G06540*	*met16*	phosphoadenylyl-sulfate reductase (thioredoxin) activity	amino acid biosynthesis	Hu et al., 2007
*AFUA_8G05630*	*chsF*	putative chitin synthase	cell wall biology	Muszkieta et al., 2014
*AFUA_5G09360*	*calA*	calcineurin a catalytic subunit	cell wall biology	Juvvadi et al., 2013
*AFUA_6G11390*	*gel2*	GPI-anchored 1,3-beta-glucanosyltransferase	cell wall biology	Mouyna et al., 2005
*AFUA_1G10380*	*nrps1*	non-ribosomal peptide synthase	cell wall biology	Reeves et al., 2006
*AFUA_6G06340*	*gfa1*	glutamine-fructose-6-phosphate transaminase activity	cell wall biology	Hu et al., 2007
*AFUA_2G13430*	*csmB*	putative chitin synthase	cell wall biology	Muszkieta et al., 2014
*AFUA_3G08520*	*rlmA*	cell wall organization, cellular response to stress	cell wall biology	Rocha et al., 2016
*AFUA_5G09580*	*rodA*	Asexual spores hydrophobin	cell wall biology	Shibuya et al., 1999
*AFUA_2G17050*	*noc3*	rRNA processing	metabolism	Hu et al., 2007
*AFUA_5G03670*	*spe2*	role in spermidine biosynthetic process	metabolism	Hu et al., 2007
*AFUA_5G03560*	*gus1*	Putative glutamyl-tRNA synthetase	metabolism	Hu et al., 2007
*AFUA_3G09020*	*pri1*	DNA primase small subunit	metabolism	Hu et al., 2007
*AFUA_2G10600*	N/A	Complex I NADH oxidoredutase	metabolism	Bromley et al., 2016
*AFUA_6G08900*	*mak5*	role in maturation of 5.8S rRNA	metabolism	Hu et al., 2007
*AFUA_3G10000*	*pkaR*	cAMP-dependent protein kinase regulatory subunit	metabolism	Zhao et al., 2006
*AFUA_4G10330*	*ramA*	role in protein farnesylation	metabolism	Norton et al., 2017
*AFUA_7G02260*	*somA*	putative role in lipid homeostasis	metabolism	Lin et al., 2015
*AFUA_7G05370*	*aspB*	Putative septin	hyphal growth	Vargas-Muñiz et al., 2015
*AFUA_6G05110*	*tom40*	role in conidium formation, hyphal growth	hyphal growth	Hu et al., 2007
*AFUA_6G09690*	*gliG*	gliotoxin production, Glutathione S-transferase	secondary metabolism	Scharf et al., 2011
*AFUA_6G09640*	*gliI*	gliotoxin production, Aminotransferase	secondary metabolism	Forseth et al., 2011
*AFUA_6G09650*	*gliJ*	gliotoxin production, Dipeptidase	secondary metabolism	Scharf et al., 2011

**Figure 5:**
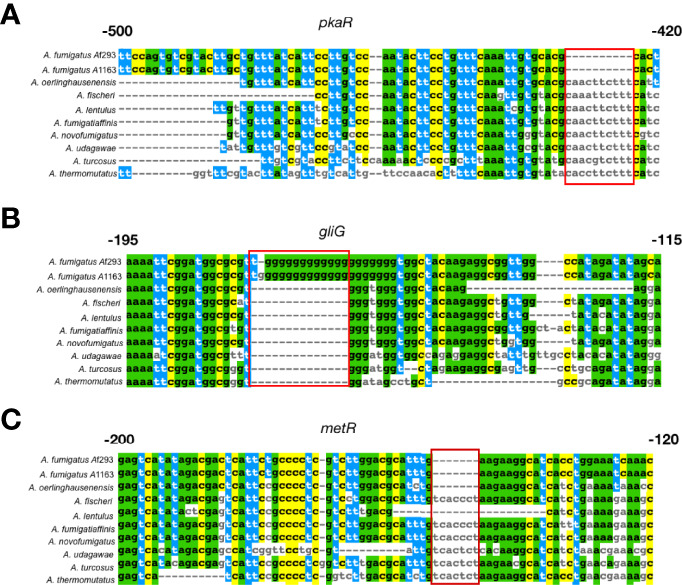
Notable examples of sequence differences between *A fumigatus* and its close relatives in non-coding regions located upstream of three known genetic determinants of *A fumigatus* virulence that exhibited signatures of a different evolutionary rate. **(A)** *pkaR* encodes a regulatory subunit involved in the regulation of the cyclic AMP – dependent protein kinase pathway; deletion of *pkaR* in *A fumigatus* has been shown to attenuate virulence in a neutropenic mouse model (Zhao et al., 2006). The sequence alignment of the non-coding region of the *pkaR* gene exhibits a 10 bp region (red box) that is uniquely deleted in *A fumigatus* but present in all other close relatives. **(B)** *gliG* encodes for a glutathione S-transferase (GST) that is part of the gliotoxin biosynthetic gene cluster and is required for gliotoxin production (Scharf et al., 2011). Gliotoxin contributes to the virulence of *A fumigatus*, inactivating host vital proteins *via* conjugation (Scharf et al., 2011). The sequence alignment of the non-coding region upstream of the *gliG* gene exhibits a 15 bp G-rich region that has been inserted in *A fumigatus* and is absent from all other species. **(C)** *metR* encodes for a TF involved in sulfur assimilation and is a known *A fumigatus* virulence factor (Amich et al., 2013). The sequence alignment of the non-coding region of the *metR* gene contains a 7 bp region that is absent only in *A fumigatus, A oerlinghausenensis*, and *A lentulus*. Colored nucleotides represent sites that are present in *A fumigatus* Af293 and shared between other species, highlighting differences between the reference strain and close relatives.

Examination of sequence alignments of non-coding regions of these 25 genes (**Table 1**) revealed several interesting patterns (**Figure 5**). For example, the non-coding region of *pkaR* exhibits a 10 bp stretch from -434 bp to -424 bp upstream of the first codon, which is deleted exclusively in *A. fumigatus* and present and largely conserved in all other species (**Figure 5A**). The non-coding region of g*fa1* also has a stretch of 5 bp exclusively deleted in *A. fumigatus* and present in all other species. Sequence alignment of the *gliG* non-coding region revealed an 11 bp G-rich insertion that is unique to the two *A. fumigatus* strains (**Figure 5B**). In addition to *A. fumigatus*-specific indels, we also observed that the non-coding regions of several genes known to be involved in *A. fumigatus* virulence exhibited indel variation across the other *Aspergillus* species examined as well. For example, the non-coding region of *metR* exhibits a 7 bp pyrimidine rich insertion that is found only in *A. fumigatus*, *A. oerlinghausenensis*, and *A. lentulus* (**Figure 5C**), while the non-coding regions of *calA* and *pri1* both have small sequences (12 bp and 5 bp, respectively) that are exclusively absent in *A. fumigatus* strains Af293 andA1163, and in *A. oerlinghausenensis.*

## Discussion

We identified a set of 732 genes whose non-coding regions were conserved between the genomes of reference strain *A. fumigatus* Af293, the more virulent *A. fumigatus* reference strain A1163, and eight closely related species. In these 732 genes, we also tested whether the branch leading to *A. fumigatus* exhibited a difference in the evolutionary rate in either its protein-coding or its non-coding regions compared to the other species. We found that the non-coding regions of 418 of these genes exhibit signatures of a different evolutionary rate in *A. fumigatus*. These 418 genes include 25 that are known genetic determinants of *A. fumigatus* virulence (Steenwyk et al., 2021b) (**Table 1**). Given the differences in reported invasive aspergillosis cases caused by *A. fumigatus* compared to other *Aspergillus* species (Steinbach et al., 2012), genetic differences in the non-coding regions of these 418 genes, and especially of these 25 genes previously connected to virulence, may play a role in varying pathogenic potentials of *Aspergillus* section *Fumigati* species.

Gene Ontology (GO) analysis of the 418 genes which exhibit signatures of a different evolutionary rate in non-coding regions revealed an enrichment for genes involved in regulation of metabolism and development. This is consistent with previous studies of the evolution of non-coding regions in humans (Haygood et al., 2007), which experienced positive selection in non-coding regions for genes involved in metabolism regulation (particularly glucose metabolism) and regulation of development (particularly the nervous system) compared to close relatives. These results raise the possibility that non-coding regions associated with particular functions in diverse taxa are more likely to experience changes in their evolutionary rates.

We compared our list of genes with signatures of different evolutionary rates with previous ChIP-seq studies of the transcription factors CrzA and SrbA, both of which are well studied genetic determinants of virulence for *A. fumigatus* (Cramer et al., 2008; Willger et al., 2008; Chung et al., 2014; de Castro et al., 2014; Colabardini et al., 2022). We found that the non-coding regions of two genes bound by CrzA (*AFUA_8G05090* and *AFUA_3G09960*) and two genes bound by SrbA (*AFUB_074100* and *AFUB_012300*) also exhibited different evolutionary rates in *A. fumigatus*. We found several nucleotide differences in these non-coding regions that likely contributed to the observed differences in evolutionary rate. Interestingly, when we examined the sequence alignments of the non-coding regions of these genes, we found differences at the TF binding sites (TFBS) between *A. fumigatus* and relatives. We hypothesize that the different evolutionary rate we observed in these *A. fumigatus* genes are due, in part, to changes in the associated TFBS, which may influence the regulation of these genes.

Comparisons between *Saccharomyces cerevisiae* and *Saccharomyces paradoxus* non-coding regions that were similar to the ones we report here, revealed that TFBS tend be more conserved in the proximal promoter region (within 200bp of the transcription start site) than the distal region, yet some differences in TFBS were reported between the two species in their respective proximal promoter regions (Schaefke et al., 2015). Evolutionary differences near the transcription start site have also been reported in *Drosophila* species, with certain species (such as *Drosophila pseudoobscura*) exhibiting an increased mutation rate upstream of the transcription start site when compared to *Drosophila melanogaster* (Main et al., 2013). Combined with the results presented here, it is likely that the evolution of non-coding regions is not uniform across closely related species and that these differences may play a functional role in downstream gene expression.

We compared our list of 418 genes with signatures of a different evolutionary rate in the non-coding regions of *A. fumigatus* to a previously curated set of 206 genetic determinants of *A. fumigatus* virulence (Steenwyk et al., 2021a) and found that 25 of the 206 exhibited a different evolutionary rate their non-coding regions between *A. fumigatus* and close relatives (**Table S6**). We found that the most represented general function amongst these 25 genes was “metabolism”, which raises the question of their impact on virulence, given the role that metabolism has been shown to play in *A. fumigatus* virulence (Willger et al., 2009). In particular, *pkaR* is essential for proper protein kinase A signaling (Griffioen and Thevelein, 2002) and plays a key role in the germination and growth of *A. fumigatus* asexual spores (Zhao et al., 2006). Moreover, *pkaR* has been shown to be required for *A. fumigatus* virulence (Lin et al., 2015) in an immunocompromised murine model of invasive aspergillosis (Fuller et al., 2011). We found that the protein-coding region of *pkaR* does not exhibit a different evolutionary rate in *A. fumigatus*, suggesting that it is conserved. However, analysis of the sequence alignment of the non-coding region revealed a 11 bp region (CAACTTCTTT) absent in *A. fumigatus* but present in all other species (**Figure 5**). Interestingly, this binding site is similar to the predicted TFBS for the *S. cerevisiae* TF Ste12 (Badis et al., 2008), a homolog to SteA in *A. fumigatus*. While it has yet to be elucidated if this region is involved in SteA binding, it may be that its absence changed the regulation of *pkaR* and thus somehow contributed to the evolution of *A. fumigatus* virulence.

The role of gliotoxin in *A. fumigatus*-mediated disease has been of increasing interest, due to its ability to inhibit the host immune response (Raffa and Keller, 2019). However, the gliotoxin biosynthetic gene cluster is found in both *A. fumigatus* and its non-pathogenic close relatives *A. oerlinghausenensis* and *A. fischeri*, and all three species are known to produce gliotoxin (Knowles et al., 2020; Steenwyk et al., 2020b). Here, we identify that three genes in the gliotoxin biosynthetic gene cluster (*gliG, gliJ, gliI*) exhibit a different evolutionary rate in their non-coding regions in *A. fumigatus*. Gliotoxin genes have been shown to require certain TFs (GliZ and RglT for example) for gliotoxin biosynthesis and/or self-protection (Schrettl et al., 2010; Ries et al., 2020; de Castro et al., 2022). Interestingly, analysis of the sequence alignment of the non-coding region of *gliG* revealed a G-rich region unique to *A. fumigatus* (**Figure 5**). G-rich regions have been previously reported to be found in biologically active sites and to play important roles in regulating cellular processes such as gene expression (Maizels and Gray, 2013; Maity et al., 2020). This *A. fumigatus*-specific G-rich region may contribute to some unknown gliotoxin expression pattern that contributes to *A. fumigatus* virulence or the lack of disease caused by other closely related *Aspergillus* species.

*metR* encodes a bZIP DNA binding protein required for sulfur metabolism in *A. fumigatus* and whose gene expression is regulated by LaeA, a major regulator of secondary metabolism (Jain et al., 2018. Pertaining to *A. fumigatus* virulence, sulfur assimilation plays key roles in oxidative stress response and gliotoxin biosynthesis (Traynor et al., 2019). Recent efforts have identified differences in the transcriptional profiles of *A. fumigatus* and relatives in response to exogenous gliotoxin, highlighting the pathways relating sulfur assimilation and gliotoxin production (de Castro et al., 2022). The non-coding region of *metR* contains a 7 bp region (TCACCT) in *A. fischeri* and five other species; in contrast, the two strains of *A. fumigatus*, *A. oerlinghausenensis* and *A. lentulu*s all lack this 7 bp motif (**Figure 5**). While it remains unclear if this 7 bp motif has a functional role in the expression of *metR*, this result nicely illustrates the complex patterns of sequence evolution of non-coding regions in this clade of pathogens and non-pathogens.

A major outstanding question emanating from our work is whether this extensive non-coding sequence variation of closely related *Aspergillus* species that vary in their pathogenicity functionally contributes to differences in gene expression between strains and species. Currently, there are no datasets available that report genome-wide differential expression data for *A. fumigatus* and close relatives; to our knowledge, the only published differential expression study of *A. fumigatus* and close relatives focused on expression differences only for genes involved in secondary metabolism (Takahashi et al., 2021). Designing and performing differential gene expression experiments in diverse *Aspergillus* species will be a future aim. Additional future work will include functionally test if the non-coding region differences we report here play a role in *A. fumigatus* expression and virulence. Further, testing if non-coding regions in a larger set of *A. fumigatus* strains exhibit differences in evolutionary rates would help to elucidate more recent evolutionary changes in *A. fumigatus* and the pathogenic differences observed in these strains as well.

## Data Availability Statement

The datasets presented in this study can be found in online repositories. The names of the repository/repositories and accession number(s) can be found in the article/**Supplementary Material**.

## Author Contributions

AB, MM, and AR designed the study and its experiments. AB performed the experiments. JS generated the species phylogeny. AB and AR wrote the manuscript. All authors commented on the manuscript and contributed to its revision.

## Funding

AB was funded by the Biological Sciences graduate program at Vanderbilt University. Research in AR’s lab is supported by grants from the National Science Foundation (DEB-1442113 and DEB-2110404), the National Institutes of Health/National Institute of Allergy and Infectious Diseases (R56AI146096 and R01AI153356), and the Burroughs Wellcome Fund. JS and AR were supported by the Howard Hughes Medical Institute through the James H. Gilliam Fellowships for Advanced Study program.

## Conflict of Interest

Author AR is a scientific consultant for LifeMine Therapeutics, Inc.

The remaining authors declare that the research was conducted in the absence of any commercial or financial relationships that could be construed as a potential conflict of interest.

## Publisher’s Note

All claims expressed in this article are solely those of the authors and do not necessarily represent those of their affiliated organizations, or those of the publisher, the editors and the reviewers. Any product that may be evaluated in this article, or claim that may be made by its manufacturer, is not guaranteed or endorsed by the publisher.
