# Supplementary material for: Extensive Non-Coding Sequence Divergence Between the Major Human Pathogen Aspergillus fumigatus and its Relatives
Source: Front Fungal Biol. 2022 Jul 7;3:802494. doi: 10.3389/ffunb.2022.802494 (PMC9977105; doi:10.3389/ffunb.2022.802494)
Supplement: Supplementary file 1 [file DataSheet_1.zip › Supplementary Figures.docx]

**Supplemental Figures and Figure Legends**


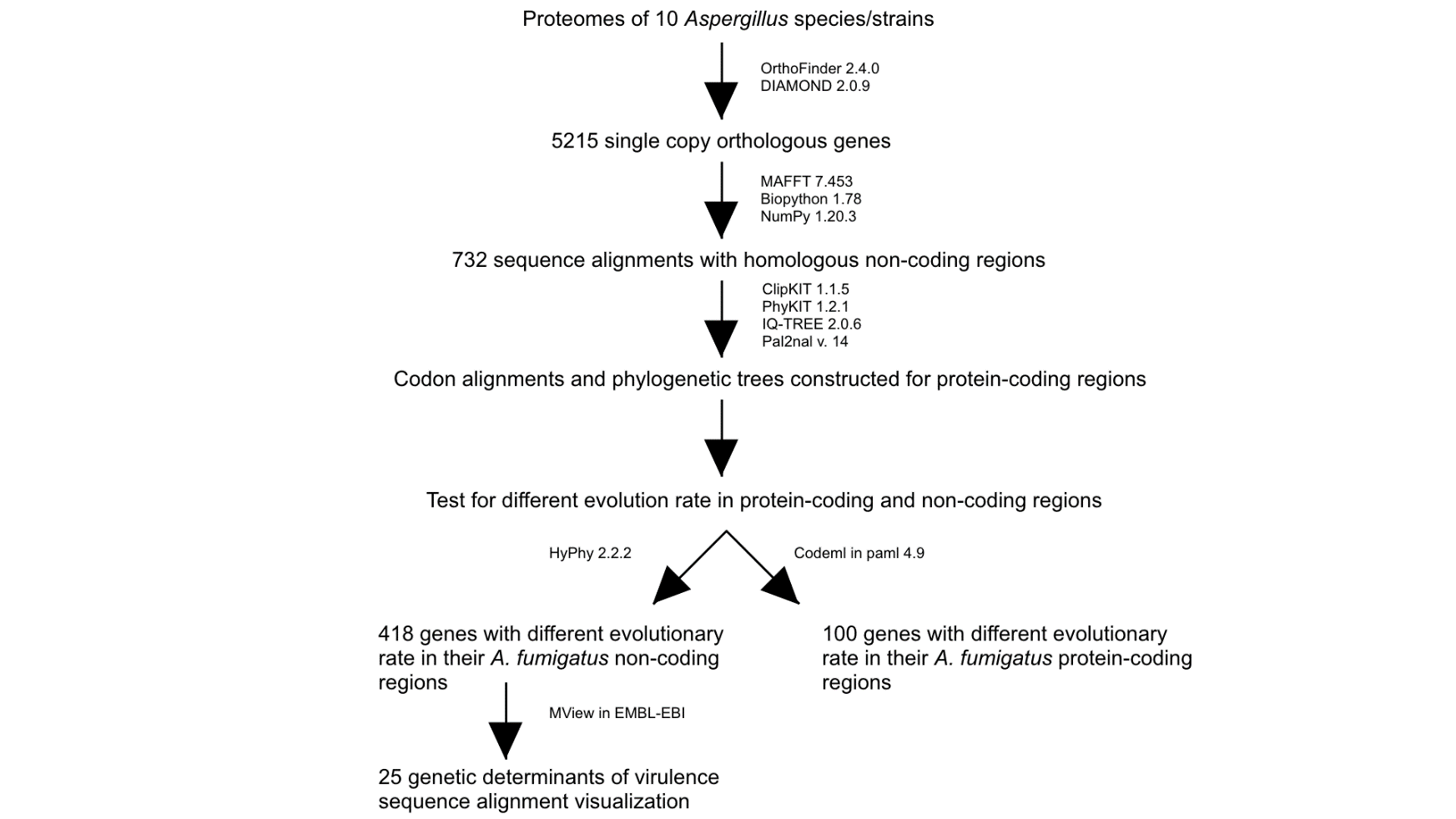


**Supplementary Figure 1. Methods Workflow.** A general outline of the steps taken to go from proteomes of 10 *Aspergillus* species to individual non-coding sequences of interest along with programs used to achieve each step of the pipeline.


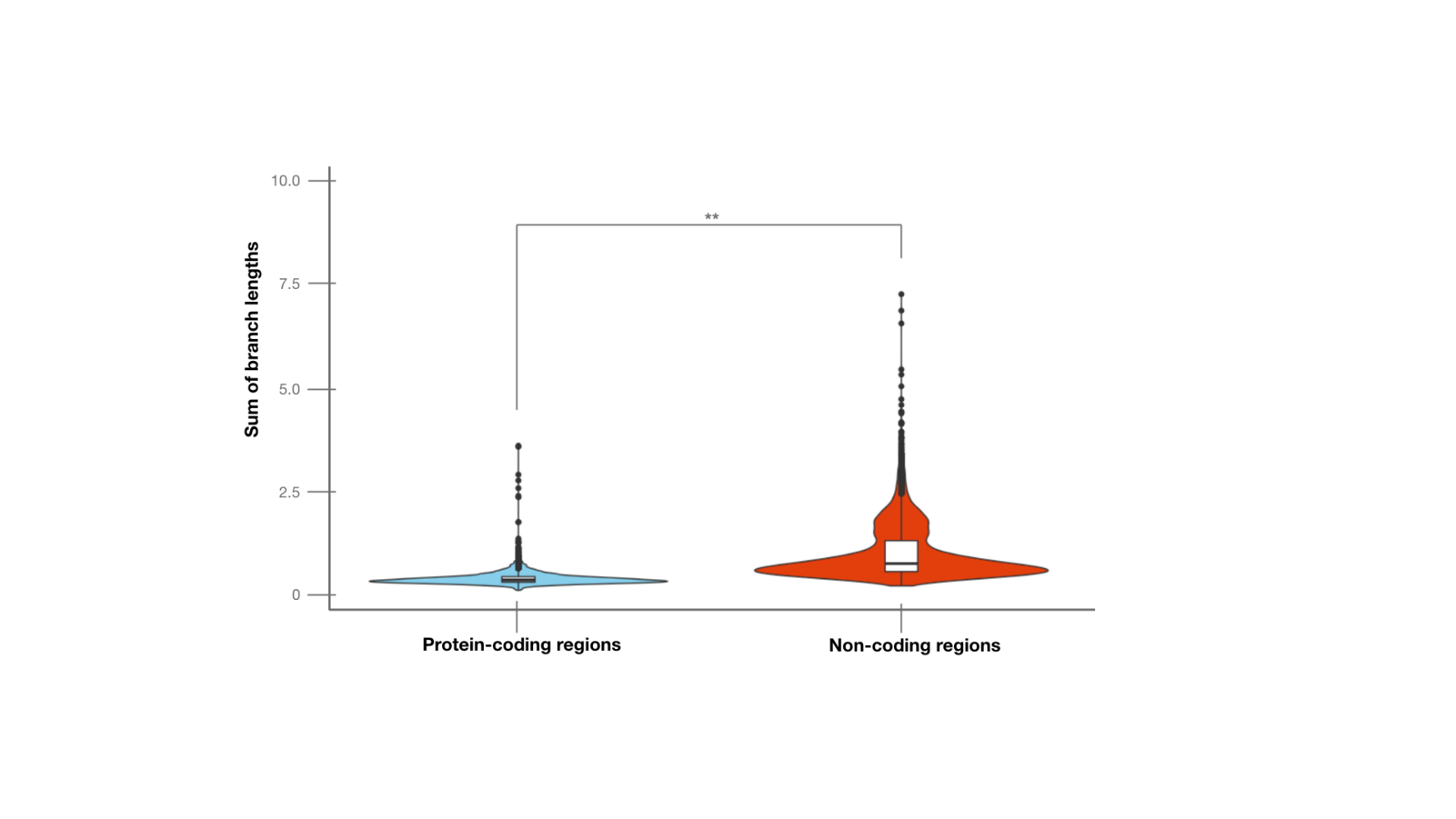


**Supplementary Figure 2. Tree Branch Lengths.** Distribution of the sum of branch lengths in gene trees from protein-coding and non-coding regions. In blue, violin plot of the sum of branch lengths for the protein-coding region trees of 5,215 single copy orthologous genes. In red, violin plot of the sum of branch lengths for non-coding region trees across 5,215 single copy orthologous genes. Non-coding region trees exhibit a significantly larger average sum of branch lengths compared to protein-coding region trees (Wilcoxon signed-ranked test; p-value = 0.004), suggesting that non-coding regions of single-copy orthologs evolve faster than protein-coding regions.


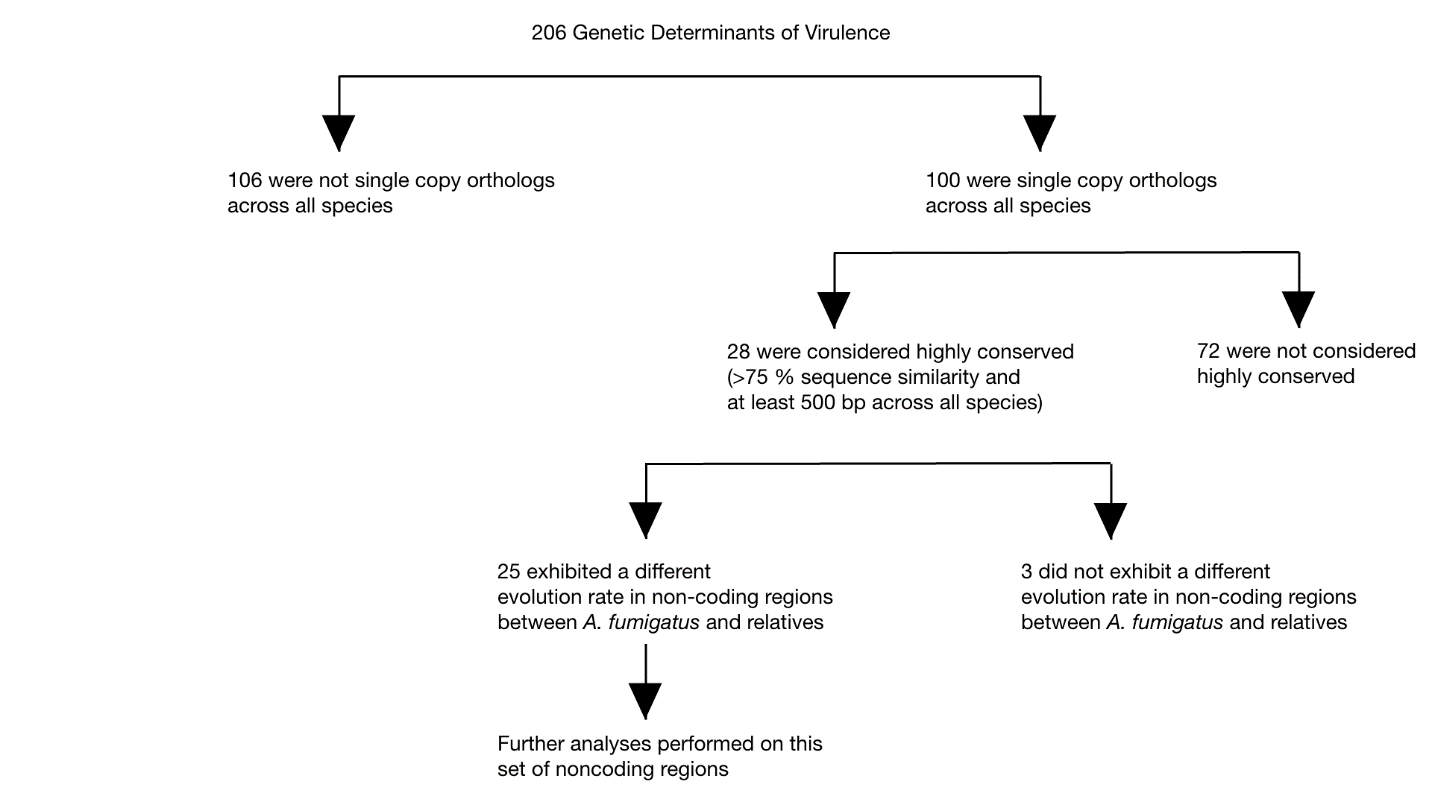


**Supplementary Figure 3. Description of protein-coding and non-coding sequence variation for 206 previously known genetic determinants of *A. fumigatus* virulence.**
